# It’s not distance but similarity of distance: changing stimulus relations affect the control of action sequences

**DOI:** 10.1007/s00426-024-01973-6

**Published:** 2024-05-11

**Authors:** Silvia Selimi, Christian Frings, Alexander Münchau, Christian Beste, Birte Moeller

**Affiliations:** 1 Department of Cognitive Psychology, Trier University, D-54286 Trier, Germany; 2 Institute of Systems Motor Science, Center of Brain, Behavior and Metabolism, Universität zu Lübeck, Lübeck, Germany; 3 Cognitive Neurophysiology, Department of Child and Adolescent Psychiatry, Faculty of Medicine, TU Dresden, Dresden, Germany

## Abstract

Interacting with our environment happens on different levels of complexity: While there are individual and simple actions like an isolated button press, most actions are more complex and involve sequences of simpler actions. The degree to which multiple simple actions are represented as one action sequence can be measured via so-called response-response binding effects. When two or more responses are executed consecutively, they are *integrated* into one representation so that repetition of one response can start *retrieval* of the other. Executing such an action sequence typically involves responding to multiple objects or stimuli. Here, we investigated whether the spatial relation of these stimuli affects action sequence execution. To that end, we varied the distance between stimuli in a response-response binding task. Stimulus distance might affect response-response binding effects in one of two ways: It might directly affect the representation of the response sequence, making integration and retrieval between responses more likely if the responses relate to close stimuli. Alternatively, the similarity of stimulus distribution during integration and retrieval might be decisive, leading to larger binding effects if stimulus distance is identical during integration and retrieval. We found stronger binding effects with constant than with changing stimulus distance, indicating that action integration and retrieval can easily affect performance also if responses refer to separated objects. However, this effect on performance is diminished by changing spatial distribution of stimuli at the times of integration and retrieval.

**Supplementary Information:**

The online version contains supplementary material available at 10.1007/s00426-024-01973-6.

## Introduction

Imagine pouring yourself a glass of water. To do so, you have to execute different actions to different objects in sequence: You have to reach for a glass, for a bottle, and then you have to open the bottle to pour. In such a sequence, we can assume that not only each given response is cognitively represented, but also the stimulus one responded to is represented with it, a phenomenon called stimulus-response binding (Henson et al., 2014; Hommel, 1998). Such stimulus-response bindings are part of short-term memory action representations that have been called event files (Hommel et al., 2001). The *Binding and Retrieval in Action Control* framework (BRAC; for an overview see, Frings et al., 2020) emphasizes two basic processes here: When responding to a stimulus, stimulus and response features are *integrated* into the event-file, so that upon repetition of one feature later on, other bound features are *retrieved* and can affect current performance. For example, if a retrieved response fits the currently appropriate response, performance is facilitated; if a retrieved response differs from the currently required one, performance is impaired. Importantly, there is evidence that similar integration and retrieval processes also occur between individual responses of an action sequence (Moeller & Frings, 2019a, b). That is, the individual event files seem to be held together by bindings between responses of the sequence (Moeller & Frings, 2021, 2022): When we execute multiple actions in sequence, the responses can be integrated into a higher-order representation of this action sequence (Moeller & Frings, 2019a, b; Selimi et al., 2022), with underlying neurophysiological processes being complex (Dilcher et al., 2021; Mielke et al., 2021; Takacs et al., 2021; Wendiggensen et al., 2022). For example, if two simple responses are executed in sequence, these are temporarily integrated. The repetition of one of the integrated responses later on can then trigger retrieval of the other response. That is, if we grab a glass and then a bottle, both responses are integrated into one action sequence representation. If we execute a previously integrated response shortly thereafter (e.g., we again reach for the bottle), the other integrated response (e.g., grabbing the glass) can be retrieved, and its execution can thus be facilitated. If the next required response does not match the retrieved response (e.g., we want to drink from the bottle instead of grabbing a glass), we would take longer to execute the response and/or make more errors. Integration and retrieval between responses (i.e., *response-response binding effects*) can be measured in a paradigm, using two prime responses followed by two probe responses, where integration is assumed for the prime responses, and retrieval is triggered by the first probe response, affecting the second probe response. Integration and retrieval then lead to specific advantages and impairments in responding: After integration of two responses (via sequential execution of them), repeating one integrated response retrieves the other, facilitating its execution and impairing the execution of other, not integrated responses (Moeller and Frings, 2019b). This effectively extends the principle of integration and retrieval that originally targeted stimulus and response features of individual actions, to individual responses within action sequences.

Presently, it is still unclear under what circumstances such higher order bindings between responses of individual event files affect further action. As many of our actions are directed towards stimuli in our environment and thus, these stimuli become part of the representation of the individual action (i.e., the event file) it seems almost imposing that stimulus relation might determine whether or not related responses are integrated to one action sequence. To the best of our knowledge, there is no previous study in which stimulus relations were examined in a response-response binding task and thus, it is yet unknown whether and how the relation of such stimuli might influence response-response binding effects.

Previous studies, focusing on integration and retrieval in individual actions indicate that especially the *spatial* relations between stimuli can affect whether they become part of the same or separate representations (van Dam & Hommel, 2010). Two clearly separable stimuli (such as an apple and a banana) became integrated into a common representation if they coincided spatially – this integration was reduced if they were spatially separated from one another (van Dam & Hommel, 2010). Moreover, when an individual response to a stimulus is executed, stimuli that are response irrelevant (i.e., distractor stimuli), can also become part of that event file (Frings et al., 2007), again depending on their spatial position in relation to the task-relevant stimulus. These distractor-response binding effects were generally stronger when stimuli were perceived as spatially connected (Frings & Rothermund, 2011; Schmalbrock et al., 2022) or spatially close (Moeller et al., 2012) than when they were farther apart. More recent findings show that integration and retrieval can be modulated separately (as emphasized in the *BRAC* framework, Frings et al., 2020). In line with this, findings regarding the spatial relation of stimuli could be further specified: Binding effects benefited from stimuli that were closely related in terms of spatial organization at the time of integration (Laub et al., 2018), while the results were mixed for the stimulus relation at the time of retrieval (Laub et al., 2018; Schmalbrock et al., 2022).

Thus, the spatial relation of stimuli seems to affect whether stimuli are represented together or separate. Findings show that this seems to be the case within an event file but could potentially also have an influence across event files in action sequences. Here we investigate this by varying the distance between the response relevant stimuli of the to-be-integrated responses and measured binding effects between these responses.

If stimulus distance plays a role in the representation of action sequences, responses given to close stimuli might elicit stronger response-response binding effects than responses given to stimuli that are positioned far apart. Yet, a different effect of stimulus distance seems likely, regarding another line of research in stimulus-response binding. In particular, characteristics of a situation have been shown to function as a sort of cue, indicating appropriateness of retrieval. For example, stimulus-response binding effects were larger if an additional task irrelevant sound was repeated from integration to retrieval than if the sound changed (Mayr et al., 2018; Qiu et al., 2022a, b; for visual stimuli, see also Frings et al., 2007). This has been interpreted as an effect of context similarity. Any present but task irrelevant (internal and external) sensations are here defined as context. Changes in these lead to a perceived context change which hinders retrieval of previously bound features. Interestingly, such modulation of binding effects is possible via the mere configuration of stimuli (e.g., the number of distractors, Laub & Frings, 2020). Consequently, the spatial relation of stimuli in the present study might not directly affect binding between responses (with larger binding effects for short than for far distances between stimuli). Rather, the *repetition* of any (close or far) spatial relation between response relevant stimuli might be a prerequisite for binding between responses to affect performance. Binding effects would only be expected if the same spatial stimulus relation is experienced during integration of responses and retrieval of one response via repetition of the other.

### The present study

To investigate whether the spatial relation of response relevant stimuli influences the representation of the respective response sequence, leading to differences in response-response binding effects, we manipulated the spatial distance between stimuli in a response-response binding paradigm (Moeller & Frings, 2019b).

In each trial of a typical response-response binding paradigm, participants give two prime responses that are followed by two probe responses. Each of these responses is indicated by a stimulus appearing on the screen with each new stimulus only appearing after execution of the previous response. It can then be assumed that upon execution of prime Response 2 the prime responses are integrated so that a repetition of one of them as the first probe response (probe R1) starts retrieval of the other, affecting performance in the second probe response (probe R2). In the current study the two prime stimuli (as well as the two probe stimuli) could appear next to each other (close) or on opposite sides of the screen (far). If the distance between stimuli becomes part of the cognitive representation of the action sequence, response-response binding effects should differ depending on the stimulus distance conditions. We manipulated stimulus distance during integration (i.e., the distance between the stimuli indicating the two prime responses) and retrieval (i.e., the distance between the stimuli indicating the two probe responses) orthogonally, resulting in four conditions: Prime close – probe close, prime far – probe far, prime close – probe far, and prime far – probe close, with the former two conditions having similar spatial stimulus relations between prime and probe and the latter two conditions having dissimilar relations. Note that each response was associated with two stimuli and the paradigm included no stimulus repetitions within a trial – that is, the binding is clearly exclusive for responses. Nevertheless, we hypothesized that the distance between response relevant stimuli in the prime and/or between response relevant stimuli in the probe might affect how responses are integrated and retrieved. Binding effects might be larger if response relevant prime stimuli are positioned close to each other than if they are positioned far from each other, indicating an effect of stimulus distance on response integration. Or the binding effect might differ for close and far probe stimuli, indicating that stimulus distance affects the response retrieval process. Finally, binding effects could be largest, if the distance between response relevant stimuli is similar during the prime (i.e., at the time of integration) and the probe (i.e., at the time of retrieval).

## Experiment

### Method

#### Participants

Effect sizes in former studies on response-response binding (computed as t/sqrt(n) were at least *d* = 0.63 (e.g., Moeller & Frings, 2019b: *d* = 0.63 and *d* = 0.88; Moeller & Frings, 2019c: *d* = 1.07; Moeller & Frings, 2019d: *d* = 0.74 and 1.07; Selimi et al., 2022: *d* = 0.98 and *d* = 0.96). A power analysis with the program G*Power assuming α = 0.05 and a power of 1–β = 0.90 suggested that at least 29 participants were necessary (Faul et al., 2007). Thirty-one students (30 women) from Trier University participated in the experiment. The samples’ median age was 22 years, with a range from 19 to 30 years. The participants were rewarded with partial course credit. One additional participant had to be excluded from the analysis due to a high number of extremely fast and erroneous responses (faster than 200ms; 380 out of 384 trials had to be discarded).

#### Design

The design included four within-subjects factors, namely, prime stimulus distance (close vs. far), probe stimulus distance (close vs. far), response R1 relation (response repetition vs. response change from prime to probe), and response R2 relation (response repetition vs. response change from prime to probe).

#### Materials and procedure

**Fig. 1:**
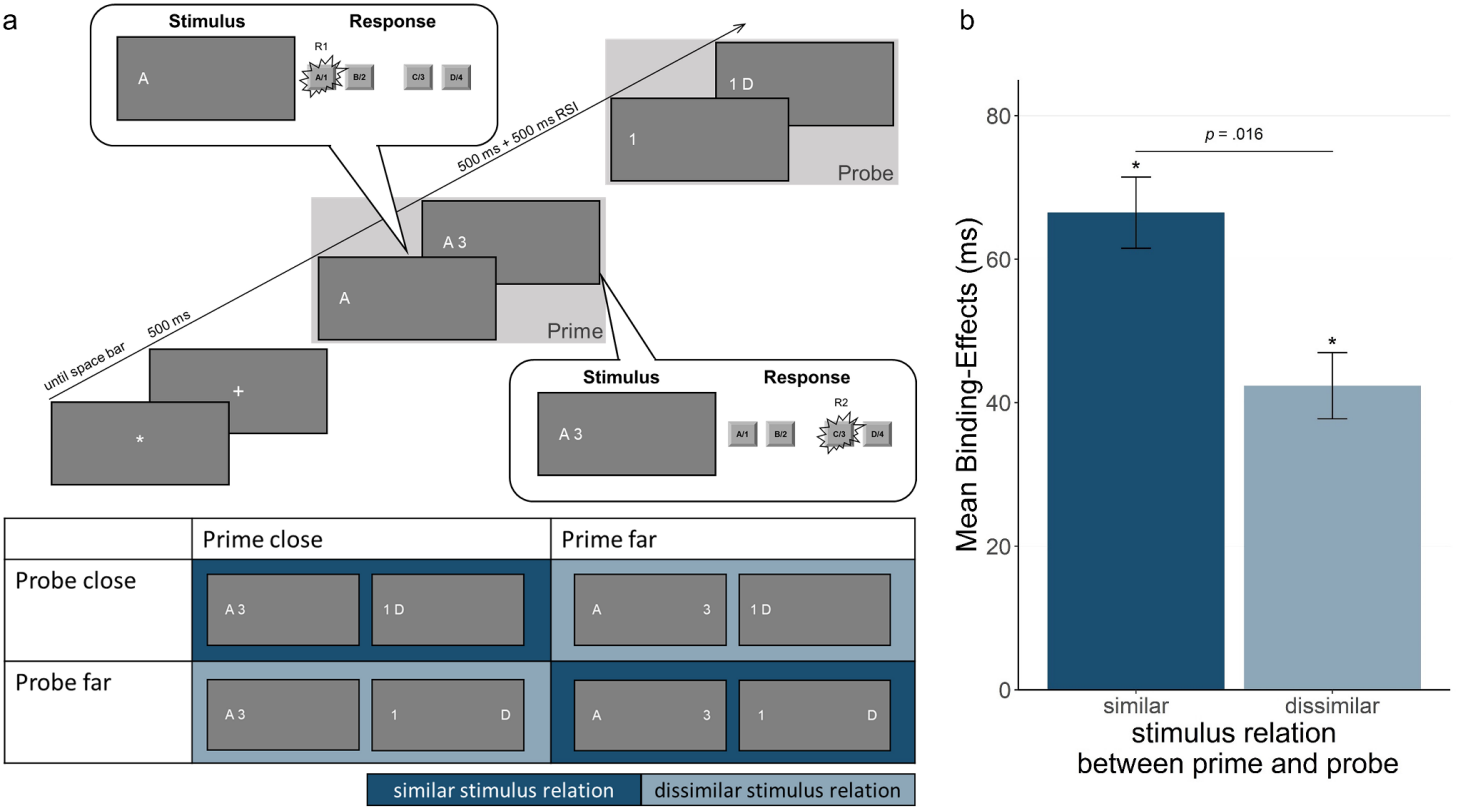
**(a)** Sequence of events in one example trial. Participants gave two successive responses, R1 and R2, both to the prime and the probe. This is an example of an R1 repetition and R2 change trial in the prime close – probe close stimuli condition. Below are stimulus positions depending on stimulus distance condition and similar vs. dissimilar stimulus relation, respectively. Close stimuli could appear either on the left (as depicted) or on the right side of the screen. The stimuli are not drawn to scale. **(b)** Mean response-response binding effects for response times as a function of stimulus relation (similar vs. dissimilar in prime and probe). Binding effects are calculated as the advantage of probe R1 repetition (vs. probe R1 change) in probe R2 repetition trials minus the advantage of probe R1 repetition (vs. probe R1 change) in probe R2 change trials: [R1cR2r - R1rR2r] - [R1cR2c - R1rR2c]. * *p* < .05 indicates whether binding effects differ significantly from zero

The experiment was programmed in PsychoPy3/PsychoJS (2021.1.2; Peirce et al., 2019) and conducted online on Pavlovia (https://pavlovia.org/). For participation, a computer with a physical keyboard was required (no tablet computers or smartphones). Instructions were presented in white [RGB: 255, 255, 255] on a grey background [RGB: 128, 128, 128]. Stimuli were the letters A, B, C, and D and the digits 1, 2, 3, and 4, each with a height of 35 pixels and presented in white. Stimuli appeared in one of four positions on the same imaginary center screen line, depending on the condition (in pixels, screen center has coordinate [0,0]: [-540, 0], [-480, 0], [480, 0] and [540,0], see Fig. 1a). For the close conditions, stimuli appeared in either the two left side or the two right side positions. That is, in half of the trials both prime stimuli and both probe stimuli appeared on the right hand side of the screen and in the other half of the trials, the stimuli appeared on the left hand side of the screen. Stimuli in the far conditions always appeared on opposite screen side positions, while maintaining a fixed distance (either [-540, 0] and [480, 0] or [-480, 0] and [540, 0]). Together, in the close and far conditions, the same number of stimuli appeared on the left and right side of the screen respectively throughout the experiment. Prime stimuli disappeared after the prime, resulting in a maximum of two stimuli visible at a time (see Fig. 1a).

#### Procedure

Before the experiment, participants gave informed consent regarding the recording of personal data and responses during the experiment and indicated their age and gender. Instructions were given on the screen. Participants were instructed to place the middle and index fingers of both hands on the keys D, F, J, and K and leave them there throughout the experiment. Each key/response corresponded to a letter and a digit (A/1: left middle finger, B/2: left index finger, C/3: right index finger, and D/4: right middle finger).

Participants’ task was to press the key corresponding to the presented letters and digits. Each trial was started by pressing the space bar while an asterisk was presented in the middle of the screen (see Fig. 1a). Then a plus sign appeared for 500 ms, followed by the first prime stimulus (letter or digit) indicating prime response R1. Immediately after a response R1 to the first prime stimulus was executed, the second prime stimulus appeared indicating prime response R2. For example, the first prime stimulus could be the letter C and the second prime stimulus the digit 1, requiring a keypress with the right index finger as prime R1 and a keypress with the left middle finger as prime R2. This was identical for both stimulus distance conditions. The position of stimuli depended on the condition (close vs. far, see Fig. 1a). After prime response R2 execution, a blank screen appeared for 500 ms and was followed by the probe. The procedure in the probe was identical to that in the prime. Every 48 trials participants were allowed to take a short break, after which they resumed the task in their own time.

The relation of R1 between prime and probe (repetition vs. change) was varied orthogonally to the relation of R2 (repetition vs. change). In R1 repetition trials (R1r), the same response was required to the stimulus indicating prime response R1 and the one indicating probe response R1. For example, if participants had to respond with their left middle finger to the first prime stimulus (e.g., A), the first probe stimulus (e.g., 1) again required a response with the left middle finger. In R1 change trials (R1c), different responses were required to the stimulus indicating prime response R1 and the one indicating probe response R1. For example, the first prime response might have required a response with the left index finger and the first probe stimulus a response with the right index finger. In R2 repetition trials (R2r), the same response was required to the stimulus indicating prime response R2 and the one indicating probe response R2. For example, the second prime stimulus indicated a response with the right index finger and the second probe stimulus also required a response with the right index finger. In R2 change trials (R2c), different responses were required to the stimulus indicating prime response R2 and the one indicating probe response R2. For example, the second prime stimulus indicated a response with the right middle finger and the second probe stimulus a response with the left middle finger. A stimulus distance manipulation was applied to the prime and probe independently, resulting in four distance conditions: prime close – probe close, prime far – probe far, prime close – probe far, and prime far – probe close, the two first ones represented a similar stimulus relation between prime and probe and the latter ones a dissimilar stimulus relation. These four distance conditions were varied block-wise with one block in each of the four conditions. For a schematic overview of all conditions, see Table A in the supplementary material. The order of blocks was balanced across participants via a Latin square. Each experimental block included 96 trials (24 of each of the four conditions R1rR2r, R1rR2c, R1cR2r, R1cR2c), resulting in 384 trials total. At the beginning of the experiment, participants passed a general practice block introducing all distance conditions (8 trials). Before an experimental block started, they practiced their task for 16 trials (a subsample of the experimental trials).

The response-response binding effect is indicated by a significant interaction of R1 relation from prime to probe and R2 relation from prime to probe: Repetition of R1 from prime to probe is assumed to start retrieval of the second prime response and thus facilitates responding in the second probe response, if R2 is repeated as well, but impairs probe R2 responding if a change of R2 is required from prime to probe. This can also be expressed as a double difference in response time or error rate performance: If (R1cR2r - R1rR2r) - (R1cR2c - R1rR2c) – in ms or percent errors – is significantly larger than zero, response-response binding effects are larger than zero (see Table B in the supplementary material for an example of the calculation). If stimulus distance directly affects response-response binding, we expect that close prime stimuli facilitate integration of responses, leading to stronger response-response binding effects with close than with far prime stimuli. Statistically, this would be indicated by the three-way interaction R1 relation × R2 relation × prime stimulus distance (i.e., an interaction of the binding effect with prime stimulus distance). If the distance of stimuli affects retrieval, this would be indicated by the three-way interaction R1 relation × R2 relation × probe stimulus distance (i.e., an interaction of the binding effect with probe stimulus distance). If the similarity of stimulus distance during integration and retrieval affects response-response binding effects, we would expect larger binding effects if stimulus distance repeats (close-close or far-far) between prime and probe than if it changes (close-far or far-close). Statistically, this would be indicated by the four-way interaction R1 relation × R2 relation × prime stimulus distance × probe stimulus distance.

### Results

The processing and analysis of data were done in R (R Core Team, 2019; version 4.2.1). We compared the experimental conditions using a repeated-measures analysis of variance (ANOVA) with type-III sums of square with the four factors R1 relation (repetition vs. change) × R2 relation (repetition vs. change) × prime stimulus distance (close vs. far) × probe stimulus distance (close vs. far). Additionally, we calculated the response-response binding effects as the advantage of probe R1 repetition (vs. probe R1 change) in probe R2 repetition trials minus the advantage of probe R1 repetition (vs. probe R1 change) in probe R2 change trials ([R1cR2r - R1rR2r] - [R1cR2c - R1rR2c]) as another way to represent the two-way interaction between response R1 relation and response R2 relation. That is, for each of the stimulus configuration conditions, the binding effect can be expressed in one number. For example, for the response times in the condition prime stimuli close/ probe stimuli close this was (664ms – 630ms) - (635ms – 672ms) = 71ms (cf. Table 1; see also Table B in the supplementary material). Also, the two factors prime stimulus distance (close vs. far) and probe stimulus distance (close vs. far) can be collapsed to the factor prime-probe stimulus relation (similar vs. dissimilar), see Fig. 1a, dark blue and light blue parts of the table, respectively. Accordingly, the critical four-way interaction can also be expressed as a *t*-test between similar vs. dissimilar prime-probe stimulus relations, with the magnitude of the binding effect as the dependent variable. Note that here *F* of the four-way interaction equals *t*² in the comparison of the binding effects. The *p*-value for the interaction is identical to the *p*-value for the t-test.

For the analysis of response times (RTs), we only included trials with correct responses R1 and R2 in both prime and probe. The error rate for prime responses (R1 or R2) was 8.9%. The probe error rates were 3.9% for R1 and 3.8% for R2 (only including trials with correct previous responses). Furthermore, we excluded RTs of more than 1.5 interquartile ranges above the third quartile of the probe R2 RT distribution of the participant (Tukey, 1977) and RTs shorter than 200 ms from the analysis. Due to these constraints, 18.5% of the trials were excluded from the RT analyses. For the mean RTs and error rates, see Table 1.

**Table 1:** Mean response times (RT in milliseconds) and mean error rates (ER in percentages) for probe responses R2, as a function of stimulus distance in prime and probe, R1 relation, and R2 relation between prime and probe

	Prime close				Prime far			
	*R2 repetition*		*R2 change*		*R2 repetition*		*R2 change*	
	*RT*	*ER*	*RT*	*ER*	*RT*	*ER*	*RT*	*ER*
Probe close								
*R1 change*	664	4.7	635	1.9	704	4.3	684	2.2
*R1 repetition*	630	3.0	672	4.2	691	2.0	703	5.0
Probe far								
*R1 change*	800	2.9	772	3.6	732	4.5	719	3.1
*R1 repetition*	765	5.4	789	5.0	685	3.5	734	6.3

The dependent variable of interest was performance in probe R2. If prime R1 and R2 are integrated, repeating prime R1 in the probe should trigger retrieval of the second prime response and thus influence performance in probe R2. In a 2 (R1 relation: repetition vs. change) × 2 (R2 relation: repetition vs. change) × 2 (prime stimulus distance: close vs. far) × 2 (probe stimulus distance: close vs. far) analysis of variance (ANOVA) on probe R2 RTs, the main effect for probe stimulus distance was significant, *F*(1, 30) = 107.81, *p* < .001, *η*_*p*_^*2*^ = 0.78, with longer RTs if probe stimuli were far apart than when they were close (*M* = 750 vs. 673 ms). The other main effects were not significant: main effect for prime stimulus distance, *F*(1, 30) = 2.89, *p* = .099, *η*_*p*_^*2*^ = 0.09, R1 relation, *F*(1, 30) = 3.13, *p* = .087, *η*_*p*_^*2*^ = 0.09, and R2 relation, *F*(1, 30) < 1, *p* = .374, *η*_*p*_^*2*^ = 0.03. Importantly, the two-way interaction of R1 and R2 relation was significant, *F*(1, 30) = 73.77, *p* < .001, *η*_*p*_^*2*^ = 0.71, indicating a general response-response binding effect. This binding effect was not modulated by prime stimulus distance, *F*(1, 30) = 1.50, *p* = .230, *η*_*p*_^*2*^ = 0.05, or probe stimulus distance, *F*(1, 30) < 1, *p* = .623, *η*_*p*_^*2*^ < 0.01, individually. However, there was a significant four-way interaction, *F*(1, 30) = 6.56, *p* = .016, *η*_*p*_^*2*^ = 0.18, which can be expressed as a *t*-test between binding effects in conditions with similar stimulus relation in prime and probe (prime close – probe close & prime far – probe far) versus dissimilar stimulus relation (prime close – probe far & probe far – prime close), that revealed a significant difference, *t*(30) = 2.56, *p* = .016, *d*_*z*_ = 0.55, with larger binding effects for similar than for dissimilar prime-probe stimulus relations (*M* = 66 vs. 42 ms, see Fig. 1b).

For the sake of completeness, the interactions between R1 relation and probe stimulus distance, *F*(1, 30) = 5.69, *p* = .024, *η*_*p*_^*2*^ = 0.16, prime and probe stimulus distance, *F*(1, 30) = 32.57, *p* < .001, *η*_*p*_^*2*^ = 0.52, and prime stimulus distance, probe stimulus distance and R2 relation, *F*(1, 30) = 6.74, *p* = .014, *η*_*p*_^*2*^ = 0.18, were also significant. All other interactions did not reach significance, *F*s < 1.6, *p*s > 0.225.

In the same analysis on error rates as the dependent variable, again the main effect of probe stimulus distance was significant, *F*(1, 30) = 5.47, *p* = .026, *η*_*p*_^*2*^ = 0.15, indicating more errors with far probe stimuli than with close stimuli (*M* = 4.3 vs. 3.4%). Additionally, the main effect for R1 relation was significant, *F*(1, 30) = 4.37, *p* = .045, *η*_*p*_^*2*^ = 0.13, while the other main effects were not, *F*s < 1, *p*s > 0.796. Again, the interaction of R1 and R2 relation was significant, *F*(1, 30) = 20.82, *p* < .001, *η*_*p*_^*2*^ = 0.41, indicating binding between the responses. This relation was further modulated by prime stimulus distance, *F*(1, 30) = 7.72, *p* = .009, *η*_*p*_^*2*^ = 0.20, but not by probe stimulus distance, *F*(1, 30) = 3.55, *p* = .069, *η*_*p*_^*2*^ = 0.11. All other interactions were not significant, *F*s < 2.1, *p*s > 0.164.

## Discussion

In this study, we investigated whether the spatial distance between stimuli becomes part of the cognitive representation of an action sequence, and thus, whether stimulus distance affects its execution. If the distance between response relevant stimuli in an action sequence is cognitively represented as part of the action sequence, this stimulus distance might modulate response-response binding effects in one of two ways: The stimulus distance might directly impact whether responses are integrated and/or retrieved. For such a direct effect we specifically expected a larger response-response binding effect for close than for far prime stimuli. Then again, similarity of stimulus distance during integration (i.e., during the prime) and retrieval (i.e., during the probe) might be crucial for binding effects to occur. This would lead to larger response-response binding effects, if the distance of response relevant stimuli was similar during integration (i.e., the prime) and retrieval (i.e., the probe) and to smaller response-response binding effects if stimulus distance changed between integration (prime) and retrieval (probe).

Our results support the latter assumption: A significant four-way interaction suggests stronger binding effects in trials with similar than with dissimilar stimulus distance between prime and probe. This is in line with previous findings in the action control literature, showing that a context can become part of the representation of individual actions, with binding effects only occurring if contexts repeat (Laub & Frings, 2020; Mayr et al., 2018; Qiu et al., 2022a, b). While such context effects are well established in the memory literature, where access to learned information is facilitated in similar as compared to dissimilar contexts (e.g., Tulving & Thomson, 1973; Zeelenberg, 2005), this is less clear for short term effects in action control. Here we found analogous effects for the repetition of stimulus distances in action sequences. Bindings between individual responses affected performance only if the distance between response relevant stimuli was identical during integration and retrieval. This might be an instance of a useful shortcut for action control in many situations: If the spatial relation of stimuli remains constant, it is more likely that the same action sequence is appropriate again, thus it makes sense that its retrieval is comparably easy. However, if sudden changes in the spatial stimulus relation occur, it is advantageous to not retrieve an action sequence, as the specific actions might not be appropriate anymore.

We need to mention that in the error data, the response-response binding effect was indeed modulated by prime stimulus distance. Yet, this interaction was not the one we predicted: we found larger binding effects for far than for close prime stimuli, rather than larger binding for close prime stimuli. Given that this effect was unpredicted, and no clear theoretical rationale supports this observed result pattern, we cannot offer a clear interpretation for the direction of the difference found. The only possible interpretation at this point is that in concert, RT and error data do not support the hypothesis that close stimulus distance at the prime enhances response-response binding effects.

It is also necessary to mention that the close and far conditions differed with regard to the distribution of stimulus-response location compatibilities across either one or two hands. If responses in one prime or probe lay on one hand, one of them was compatible and one was incompatible with its stimulus location, respectively in the far condition, while both responses were either compatible or incompatible with their according stimulus location in the close condition. Importantly, the number of compatible and incompatible responses was identical in the close and far conditions, overall and also for each hand. It was merely the distribution of spatially (in)compatible responses on either one or two hands that differed between conditions. While we cannot be sure how these compatibilities affected response times and error rates and whether that led to differences between the two conditions (close and far), we do not see how they could have affected the response-response binding effect. Since all of the four conditions, necessary to calculate the binding effect had the same probability of including stimuli, mapped to one or to two hands, all conditions were equally affected by these compatibilities. That is, the difference in (in)compatibility distribution on one vs. two hands might have led to a difference in mean RTs or ERs between the close and far conditions, but not to differences between the binding effects.

Note that shifts of attention (that might be indicated by the main effect for probe stimulus distance) could have contributed to the observed pattern. In particular, attention shifts may become integrated into the representation of an action sequence. In fact, these may function like another response that may repeat or change (in the case of overt shifts of attention; see Schöpper et al., 2022) or even as a more abstract control parameter that binds to the context (Dignath et al., 2019; Dignath & Kiesel, 2021; Egner, 2014). In the present study, conditions with similar stimulus distances in prime and probe always or never had an obligatory shift of attention within the prime/probe episode (i.e., the necessity for an attentional shift always repeated). Conditions with dissimilar stimulus distances had such an obligatory shift only in one of the two episodes. Thus, attentional shifts might offer a somewhat more fine-grained but not contradicting explanation to the results, with benefits for binding effects upon repetition, but impairment upon changes regarding attentional shifts.

Results suggest that stimulus distance did not directly influence the relation of two event files. In fact, responses were integrated and retrieved with close as well as with far stimuli. Thus, multiple responses seem to be represented as one action sequence regardless of stimulus distance. In this, results differ from findings in individual actions. There, the spatial relation between multiple stimuli altered whether they were represented as part of the same event file, with spatially close or connected stimuli being represented together in one event file, while spatially separated stimuli were not (Frings & Rothermund, 2011; Moeller et al., 2012; Schmalbrock et al., 2022). Spatial stimulus relations seem to influence the occurrence of binding and retrieval on the level of individual event files, but not across event files.

Note that it makes sense that responses are represented in one action sequence, regardless of whether stimuli are spatially farther apart or closer together, as we are usually quite flexible in adapting our movements according to our surroundings (e.g., Gallivan et al., 2018), so that small stimulus distances (as in this study) may not be particularly relevant for the execution of the action sequence. Yet, it is possible that a more extreme manipulation with greater stimulus distances would have directly influenced binding effects.

It has been shown that distances between responses systematically influence the representation of these responses (see e.g., Lakens et al., 2011; Nett & Frings, 2014). Therefore, it was important to measure the effect of variation in stimulus distance without potential influences of response distance. We ensured this by using identical response locations in all conditions. While this is representative for some everyday actions like turning on the television by using a remote control, stimulus and response location are related in many other everyday actions (e.g., we often have to touch stimuli to interact with them). The independence of stimulus and response location in the present study may have made stimulus distance even less relevant, as participants did not need to adjust their actions to accommodate for the stimulus distance.

As mentioned before, we could show that stimulus distance did not directly modulate representation of responses to an extent that response-response binding effects were affected (neither prime stimulus distance nor probe stimulus distance modulated the binding effect as predicted). Notably, there are findings showing that the spatial relation of possible *responses* can have an influence on the perceived relation of *stimuli*: If responses were separated via response keys, this facilitated the discrimination of corresponding stimulus features (Lakens et al., 2011; Nett & Frings, 2014; but see Schäfer & Frings, 2021). Apparently, the representation of stimuli is affected by spatial response relations, but the representation of multiple responses is not affected by spatial stimulus relations. This asymmetry is in line with existing research (e.g., Schäfer & Frings, 2021). In addition, while modulating the spatiotemporal relation of responses directly affected how strongly they are integrated and/or retrieved (Fournier & Gallimore, 2013), binding between responses was not influenced by the spatiotemporal relation of stimuli (see also Fournier & Gallimore, 2013) or the spatial relation of visual effects elicited by responses (Selimi et al., 2022). Thus, response-response bindings seem to be somewhat robust to influences of spatial (and potentially also temporal, see Fournier & Gallimore, 2013) stimulus relation within the current episode.

In conclusion, our results indicate that integration and retrieval between responses are not directly influenced by the spatial distribution of the acted upon stimuli. The basic action control mechanisms of feature integration and retrieval function equally well in response sequences, referring to closely spaced stimuli and response sequences, referring to widely separated stimuli or objects. Yet, response-response binding effects are larger if stimulus distance was identical at the times of integration and retrieval. That is, it is not the stimulus distance per se that affects binding and retrieval between responses. Yet, a *change* in the characteristics of stimulus spacing between integration and retrieval seems to diminish the effect of integration and retrieval mechanisms on ongoing performance in response sequences.

### Electronic supplementary material

Below is the link to the electronic supplementary material.

Supplementary Material 1

## Data Availability

All data generated and/or analyzed during the current study is available in the PsychArchives repository under: 10.23668/psycharchives.12199. The experiment was not preregistered.
