# Supplementary material for: It’s not distance but similarity of distance: changing stimulus relations affect the control of action sequences
Source: Psychol Res. 2024 May 11;88(5):1727–36. doi: 10.1007/s00426-024-01973-6 (PMC11281967; doi:10.1007/s00426-024-01973-6)
Supplement: Supplementary file 1 — Supplementary Material 1 [file 426_2024_1973_MOESM1_ESM.docx]

***Supplementary Information***

**Table A.** *Schematic overview of the conditions*

|  | Prime close | | | |  | | Prime far | | | |
| --- | --- | --- | --- | --- | --- | --- | --- | --- | --- | --- |
|  | *R2 repetition* | | *R2 change* | |  | *R2 repetition* | | | *R2 change* | |
| Probe close |  |  |  |  |  |  | |  |  |  |
| *R1 change* | R1cR2r, prime close, probe close | | R1cR2c, prime close, probe close | |  | R1cR2r, prime far, probe close | | | R1cR2c, prime far, probe close | |
| *R1 repetition* | R1rR2r, prime close, probe close | | R1rR2c, prime close, probe close | |  | R1rR2r, prime far, probe close | | | R1rR2c, prime far, probe close | |
| Probe far |  |  |  |  |  |  | |  |  |  |
| *R1 change* | R1cR2r, prime close, probe far | | R1cR2c, prime close, probe far | |  | R1cR2r, prime far, probe far | | | R1cR2c, prime far, probe far | |
| *R1 repetition* | R1rR2r, prime close, probe far | | R1rR2c, prime close, probe far | |  | R1rR2r, prime far, probe far | | | R1rR2c, prime far, probe far | |

*Note.* R1: response 1, R2: response 2, c: response identity change, r: response identity repetition; dependent variables were response times and error rates. Dark blue indicates similar stimulus distance in prime and probe, light blue indicates dissimilar stimulus distance in prime and probe.

**Table B**. *Example for calculation of the binding effect*

|  | *R2 repetition* | *R2 change* |
| --- | --- | --- |
| *R1 change* | 664 | 635 |
| *R1 repetition* | 630 | 672 |
| *Priming Effect* | 664 – 630 = 34 | 635 – 672 = -37 |
| *Binding Effect* | 34 - (-37) = 71 | |

*Note*. Priming of probe R2 by repetition of R1 from the prime (R1 change minus R1 repetition) is calculated for R2 repetition and R2 change conditions. The difference between these priming effects is the binding effect: Priming of R2 is only beneficial if the primed response repeats. It impairs performance, if a different than the primed response is required. Response times in ms, used in the example are from the prime close, probe close condition.
